# Dietary Protein and Physical Activity Interventions to Support Muscle Maintenance in End-Stage Renal Disease Patients on Hemodialysis

**DOI:** 10.3390/nu11122972

**Published:** 2019-12-05

**Authors:** Floris K. Hendriks, Joey S. J. Smeets, Frank M. van der Sande, Jeroen P. Kooman, Luc J. C. van Loon

**Affiliations:** 1 Department of Human Biology, NUTRIM School of Nutrition and Translational Research in Metabolism, Maastricht University Medical Centre+, P.O. Box 616, 6200 MD Maastricht, The Netherlands; f.hendriks@maastrichtuniversity.nl (F.K.H.);; 2 Department of Internal Medicine, NUTRIM School of Nutrition and Translational Research in Metabolism, Maastricht University Medical Centre+, P.O. Box 616, 6200 MD Maastricht, The Netherlands; jeroen.kooman@mumc.nl; 3 Division of Nephrology, Department of Internal Medicine, Maastricht University Medical Centre+, P.O. Box 5800, 6202 AZ Maastricht, The Netherlands

**Keywords:** muscle wasting, exercise, nutrition, kidney disease

## Abstract

End-stage renal disease patients have insufficient renal clearance capacity left to adequately excrete metabolic waste products. Hemodialysis (HD) is often employed to partially replace renal clearance in these patients. However, skeletal muscle mass and strength start to decline at an accelerated rate after initiation of chronic HD therapy. An essential anabolic stimulus to allow muscle maintenance is dietary protein ingestion. Chronic HD patients generally fail to achieve recommended protein intake levels, in particular on dialysis days. Besides a low protein intake on dialysis days, the protein equivalent of a meal is extracted from the circulation during HD. Apart from protein ingestion, physical activity is essential to allow muscle maintenance. Unfortunately, most chronic HD patients have a sedentary lifestyle. Yet, physical activity and nutritional interventions to support muscle maintenance are generally not implemented in routine patient care. To support muscle maintenance in chronic HD patients, quantity and timing of protein intake should be optimized, in particular throughout dialysis days. Furthermore, implementing physical activity either during or between HD sessions may improve the muscle protein synthetic response to protein ingestion. A well-orchestrated combination of physical activity and nutritional interventions will be instrumental to preserve muscle mass in chronic HD patients.

## 1. Introduction

Chronic kidney disease (CKD) is currently a public health problem with a global prevalence of 10% and the cause of approximately 33 million disability-adjusted life-years worldwide [1,2]. Development and progression of CKD are associated with the age-related decline in renal function, especially in individuals with hypertension and diabetes mellitus [3,4,5]. Therefore, the rapid ageing of our population is expected to further increase prevalence of CKD and its progression to end-stage renal disease (ESRD) [6,7]. The glomerular filtration rate in ESRD patients is below 15 mL/min/1.73 m^2^ and insufficient to adequately remove metabolic waste products and fluids from the body [8,9]. Due to the accumulation of metabolic waste products in their body, ESRD patients experience phenotypic changes that resemble the ageing process, with a progressive loss of skeletal muscle mass and strength [10].

To prevent lethal consequences of metabolic waste product accumulation in ESRD patients, hemodialysis (HD) can be used to partially replace renal solute removal [11]. Over the past decades, survival of patients undergoing HD has improved substantially [12,13]. However, prevention of the adverse effects of HD on body composition has made less progression. After initiation of chronic hemodialysis (CHD) therapy, the age-related loss of skeletal muscle mass and strength accelerates and patients typically develop impairments in physical function [14,15,16,17]. Protein-energy wasting, a severe state of malnutrition, is observed in 28%–54% of CHD patients [18,19]. Loss of skeletal muscle mass and strength predisposes CHD patients to frailty and substantially reduces their quality of life [20]. Furthermore, the decline in skeletal muscle mass and strength is associated with higher hospitalization and mortality rates in CHD patients [20,21,22]. As the duration of CHD treatment is associated with its detrimental effects on body composition, the improved survival rate of CHD patients will generate new challenges for healthcare [14]. This emphasizes the need to understand and counteract skeletal muscle mass and strength loss in CHD patients.

## 2. Muscle Maintenance

Skeletal muscle mass is regulated through a dynamic balance between continuous synthesis and breakdown of muscle proteins. The muscle protein pool has shown to possess a turnover rate of 1%–2% per day, allowing skeletal muscle tissue to adapt to circumstances such as changes in physical activity pattern (e.g., muscle hypertrophy following resistance-type exercise training) [23]. Ingesting several protein-containing meals throughout the day results in a sinusoidal pattern of subsequent increases and decreases in skeletal muscle protein synthesis and breakdown rates [24]. Skeletal muscle protein synthesis rates are high during post-prandial periods and low during post-absorptive periods, whilst skeletal muscle protein breakdown rates follow a reverse pattern. Muscle maintenance is achieved when skeletal muscle protein synthesis rates equal skeletal muscle protein breakdown rates over a given period.

Protein ingestion is an essential requirement to maintain skeletal muscle mass. After consumption, dietary protein is absorbed as amino acids in the intestine, with a large fraction being subsequently released into the circulation [25]. The release of amino acids into the circulation following protein ingestion elevates plasma amino acid concentrations for a post-prandial period of up to 5 h [26]. These circulating plasma amino acids serve as precursors for de novo synthesis of muscle protein [27]. However, amino acids are more than simply building blocks for muscle protein synthesis, as they can function as signaling molecules. The post-prandial increase in plasma essential amino acid concentrations, and leucine in particular, stimulates anabolic signaling through several molecular pathways, such as the mammalian target of rapamycin complex 1 (mTORC1) pathway [28,29]. This post-prandial anabolic signaling increases skeletal muscle protein synthesis rates and inhibits proteolysis, allowing net muscle protein accretion [27].

Muscle loss can be attributed both to an increase in muscle protein breakdown as well as to a decline in muscle protein synthesis rates. Previous work has reported increased muscle proteolysis in CHD patients due to inflammation, metabolic acidosis, and the dialysis procedure itself [30,31,32,33]. Furthermore, it has been suggested that the muscle protein synthetic response to feeding is impaired in patients with CKD [34]. Whereas a maximal post-prandial muscle protein synthetic response has been reported after ingesting up to 20 g of a high-quality protein in healthy young adults, a lesser response has been observed in older individuals [27,35,36]. More recently, van Vliet et al. were unable to detect a measurable increase in skeletal muscle protein synthesis rates in CHD patients following ingestion of a meal containing 20 g protein [37]. The latter suggests that CHD patients suffer from a blunted muscle protein synthetic response to feeding, a phenomenon that has been coined anabolic resistance. In healthy elderly individuals, it has been shown that the anabolic resistance of skeletal muscle tissue can be overcome through ingesting a greater amount of protein (at least 30 g of a high-quality protein) [38] and/or performing a bout of resistance-type exercise prior to feeding [39]. When tailored specific to CHD patients, these anabolic strategies may prove essential to attenuate or even prevent the accelerated loss of skeletal muscle mass and strength in ESRD patients undergoing HD.

## 3. Dietary Protein Intake in ESRD Patients on HD

For healthy young adults, the recommended dietary protein intake to achieve a net balance between muscle protein synthesis and breakdown rates has been set at 0.8 g protein/kg body weight/day by the World Health Organization [40,41]. This level of protein intake may not be sufficient to support muscle maintenance in CHD patients. According to the National Kidney Foundation K/DOQI Clinical Practice Guidelines, these patients are recommended to ingest >1.2 g protein/kg body weight/day [42,43,44,45]. However, CHD patients generally do not meet this recommended level of protein intake. Previous studies in this population have observed a dietary protein intake of 0.9–1.0 g protein/kg body weight/day [46,47,48,49,50,51]. Especially on dialysis days, factors such as time constraints and reduced appetite make it difficult for patients to consume ample dietary protein [52]. As a result, dietary protein intake in CHD patients has been reported to be ~0.8 g protein/kg body weight on dialysis days compared to ~1.0 g protein/kg body weight on non-dialysis days [50].

In addition to low protein intake, another factor compromises plasma amino acid availability on dialysis days. During HD, both metabolic waste products as well as circulating amino acids are able to diffuse through the semipermeable dialysis membrane [11]. The diffusion into the dialysate results in a considerable extraction of circulating amino acids throughout HD [30,53,54,55,56]. We have recently shown that during a single HD session, ~12 g amino acids are extracted from the circulation in CHD patients who ingest their habitual diet during HD [57]. This amount equals the quantity of amino acids that is released into the circulation following ingestion of a typical meal (containing 20–25 g protein). Loss of circulating amino acids causes a significant decline of plasma amino acid concentrations throughout HD [55,57]. Moreover, Ikizler et al. showed that in fasting CHD patients, plasma amino acid concentrations remain low for at least 2 h after cessation of HD [30]. The HD-induced decline in plasma amino acid concentrations has been shown to cause substantial catabolism of skeletal muscle tissue in fasted CHD patients [58,59]. The continuous extraction of amino acids throughout HD stimulates skeletal muscle tissue to release amino acids into the circulation [60,61]. This homeostatic process attenuates the decline in plasma amino acid concentrations and may prevent subsequent detrimental effects on organs that are necessary to sustain life [62]. In addition, the decline in plasma amino acid concentrations reduces the availability of precursors for de novo synthesis of muscle proteins during and following HD. To allow a muscle protein synthetic response during this period, the extraction of circulating amino acids should be compensated for through amino acid and/or protein administration.

**Figure 1:**
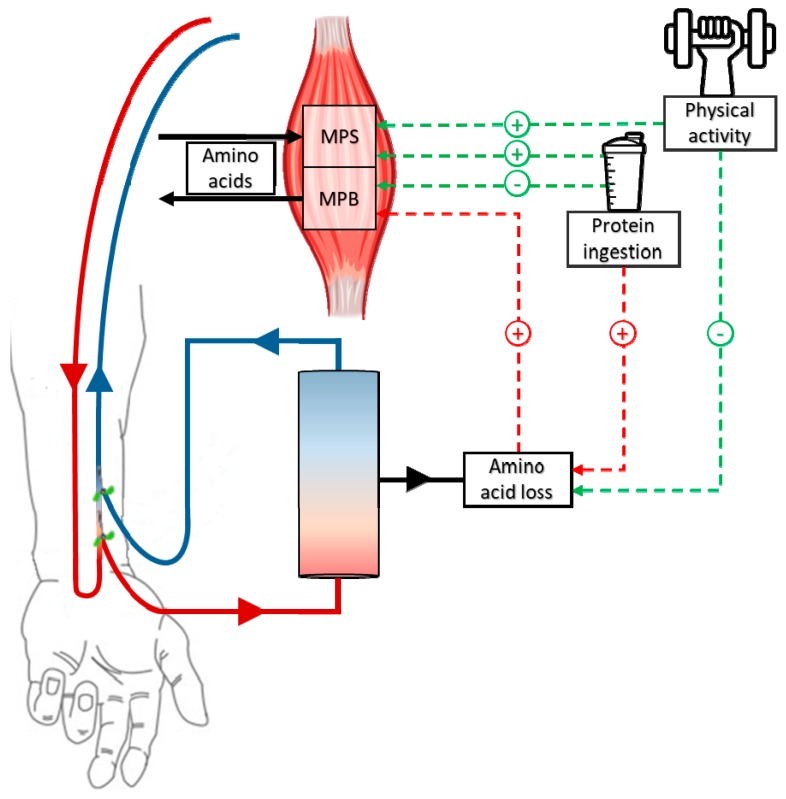
Conceptual overview of the effects of hemodialysis, protein ingestion, and physical activity on the muscle protein synthetic and proteolytic response. The extraction of amino acids during hemodialysis (HD) stimulates muscle protein breakdown (MPB) rates due to decreased plasma amino acid concentrations. Protein ingestion can maintain, or even increase, plasma amino acid concentrations throughout HD, which increases muscle protein synthesis (MPS) rates, while it may attenuate the HD-induced increase in MPB rates. However, elevated plasma amino acid concentrations also increase the amount of amino acids that are extracted during HD. Physical activity before or during HD may increase the use of plasma amino acids for de novo MPS, and thereby reduce the amount of amino acids that are extracted from the circulation during HD. Dashed lines in green represent processes that support muscle maintenance, whereas dashed lines in red represent processes that compromise muscle maintenance.

Provision of protein-rich nutrition during HD is often recommended to increase dietary protein intake on dialysis days [63,64,65,66]. Ingestion of 40–60 g protein has been shown to prevent the HD-induced decline in plasma amino acid concentrations in multiple studies [58,59,67,68]. Furthermore, Pupim et al. demonstrated that ingestion of 57 g protein resulted in a positive forearm amino acid balance throughout HD [58]. Thus, HD-associated skeletal muscle catabolism may be prevented through ingestion of sufficient protein during HD. Several studies have also observed long-term beneficial effects of protein supplementation during HD, such as an increase in lean body mass, improvement in physical function, and decrease in mortality [69,70,71]. However, data from our lab [57] and others [56,67] indicate that protein ingestion during HD is also accompanied by an increase in amino acid extraction, presumably due to a higher subsequent plasma-dialysate diffusion gradient (Figure 1). Due to this extraction following protein ingestion during HD, less amino acids become available to stimulate muscle protein synthesis rates and serve as precursors for de novo synthesis of muscle protein. Considering the anabolic resistance of skeletal muscle tissue that is also present in this population, CHD patients will need to ingest well above 20 g high-quality protein during HD to allow a post-prandial increase in skeletal muscle protein synthesis and an inhibition of proteolysis.

However, high quality (animal-derived) protein is rich in phosphorous [72]. In CHD patients, an increased dietary protein intake may lead to hyperphosphatemia or the need for phosphate binders. Furthermore, it has been suggested that an increased dietary protein intake in CHD patients provides more uremic toxin precursors and leads to higher uremic solute concentrations between HD sessions [73]. Recently, our laboratory has shown that the ingestion of branched-chain ketoacids, which contain no phosphorous or nitrogen, significantly stimulates skeletal muscle protein synthesis rates in healthy elderly individuals [74]. Ketoacid supplementation in CKD patients has been shown to reduce the generation of toxic metabolic waste products, while maintaining a good nutritional status [75]. However, it remains to be established whether ketoacid supplementation could support muscle maintenance in CHD patients.

## 4. Physical Activity in ESRD Patients on HD

Another key component for muscle maintenance is physical activity. Physical activity and exercise stimulate skeletal muscle protein synthesis rates, with post-absorptive muscle protein synthesis rates being elevated for up to 24 or even 48 h [76,77]. Furthermore, physical activity performed prior to food intake augments the post-prandial muscle protein synthetic response to feeding [78,79,80,81]. In contrast, a decline in physical activity reduces the muscle protein synthetic response to feeding [82,83,84]. In other words, whereas physical activity makes skeletal muscle tissue more sensitive to the anabolic properties of amino acids, muscle disuse leads to anabolic resistance of skeletal muscle tissue [85]. In support, daily exercise has been shown to increase skeletal muscle protein synthesis rates throughout the day [86], while a decline in physical activity has been shown to lower daily muscle protein synthesis rates [87]. Consequently, ample physical activity has been associated with a reduced age-related loss of muscle mass and strength [88,89], whereas a decline in the level of physical activity (e.g., during bed rest or limb immobilization) has been shown to induce a rapid decline in muscle mass and strength [90,91].

According to the Physical Activity Guidelines for Americans, patients with chronic diseases should follow the key physical activity guidelines for healthy adults to achieve substantial health benefits [92]. These guidelines recommend patients to perform at least 150–300 min per week of moderate-intensity aerobic exercise, 75–150 min of vigorous-intensity aerobic exercise per week, or an equivalent combination of both. In addition, muscle-strengthening activities that involve all major muscle groups should be performed at least twice per week. However, these guidelines do not contain specific recommendations for CHD patients. The Renal Association Clinical Practice Guideline on Hemodialysis recommends that all CHD patients without contraindication should perform at least 30 min of supervised moderate-intensity exercise during every dialysis session [93]. In addition, the guideline states that CHD patients should be encouraged to undertake physical activity on non-dialysis days. In line with this recommendation, it has recently been suggested that mortality rates are reduced in CHD patients who perform at least 4000 steps on non-dialysis days [94].

However, CHD patients typically adopt a sedentary lifestyle and spend less time being physically active than healthy adults [95,96]. In the United States, almost 50% of CHD patients perform exercise once or less than once per week [96]. A HD session represents a long (3–4 h) sedentary period, which often hinders CHD patients to engage in physical activity and, as such, dialysis treatments contribute to the lower physical activity levels [97,98]. Gomes et al. observed that CHD patients took 4362 ± 2084 and 7007 ± 3437 steps on dialysis and non-dialysis days, respectively, compared to 8792 ± 2870 steps taken by age-matched healthy controls [98]. The low habitual physical activity level in these patients is another key factor responsible for the accelerated loss of muscle mass and strength in CHD patients [17]. Interventions in CHD patients targeted to preserve or even increase muscle mass should not only provide nutritional support but also increase physical activity levels to maximize their impact.

## 5. Interventions to Support Muscle Maintenance in ESRD Patients on HD

Physical activity interventions for CHD patients may implement exercise during HD (intradialytic) or between HD sessions (interdialytic). A recent meta-analysis by Clarckson et al. reported no differences in the efficacy of intradialytic when compared with interdialytic exercise on improvements of physical function in CHD patients [99]. Due to exercise intolerance, CHD patients typically show low adherence and poor compliance to long-term unsupervised physical activity intervention programs [100]. HD sessions represent an opportunity to integrate supervised physical activity in the weekly routine of CHD patients. Intradialytic physical activity is considered safe and shows greater adherence rates than interdialytic physical activity [100,101,102]. Furthermore, supervision of intradialytic exercise sessions provides the opportunity to prescribe a patient-specific and progressive exercise program. Physical activity during HD has some limitations compared to interdialytic physical activity, such as constrains regarding exercise intensity and upper limb exercises. On the other hand, intradialytic physical activity provides distraction for CHD patients during their treatment and has been shown to improve their quality of life [101]. Therefore, we would advocate the implementation of an intradialytic exercise program in lifestyle interventions designed for (sedentary) CHD patients.

In addition to timing, the type of exercise is an important determinant of its potential to support muscle maintenance. Resistance-type exercise training is considered most potent to augment muscle mass and strength. In healthy adults, resistance-type exercise training has been shown to induce a robust increase in both skeletal muscle mass as well as strength [103,104,105]. Furthermore, resistance-type exercise also sensitizes skeletal muscle tissue to the anabolic properties of amino acids and, as such, increases the post-prandial muscle protein synthetic response to feeding [78,79,81]. In support, it has been reported that a single bout of resistance-type exercise performed prior to HD increases amino acid uptake by muscle tissue following intradialytic protein ingestion [106]. Intradialytic resistance-type exercise programs have shown to increase skeletal muscle strength, thereby improving physical function outcome measures such as the 6-min walk test [99,107,108,109,110]. In a systematic review of nine trials that assessed progressive resistance-type exercise training in ESRD patients on HD, Chan and Cheema concluded that resistance-type exercise training can effectively induce regional skeletal muscle hypertrophy [111]. However, due to inconsistent results of previous studies [69,112,113,114,115,116,117,118], it remains unclear whether resistance-type exercise can increase skeletal muscle mass on a whole-body level in CHD patients.

Protein ingestion during recovery from resistance-type exercise is required to achieve a positive net protein balance and, as such, to allow net muscle protein accretion [76]. Due to practical matters, the majority of studies that assessed the impact of resistance-type exercise training in CHD patients implemented their training sessions before or during HD [119]. As circulating amino acids are extracted during HD, recovery from those exercise sessions typically occurred during conditions of reduced amino acid availability. This may have attenuated the anabolic effects of the exercise training programs. Furthermore, the combination of amino acid extraction during HD and the anabolic resistance of skeletal muscle tissue in CHD patients likely increases the amount of protein that is required following intradialytic resistance-type exercise. We suggest that at least 30 g protein should be provided to CHD patients during recovery from resistance-type exercise performed immediately prior or during HD to allow a muscle protein synthetic response.

Besides protein ingestion during recovery from exercise, it has been advocated that every main meal (breakfast, lunch, and dinner) should contain 20 g high-quality protein to optimally stimulate muscle protein synthesis rates throughout the day [120,121]. We suggest that CHD patients should ingest well above 20 g high-quality protein per main meal to compensate for the blunted muscle protein synthetic response to feeding, recognizing that additional measures to prevent hyperphosphatemia might be necessary. In addition, ingesting a protein-rich snack prior to sleep, especially on training days, may further support muscle mass maintenance [24]. Though the impact of these nutritional strategies has not been assessed in CHD patients, they would likely be supplemental in the prevention of protein malnutrition in this population. Effectiveness of any nutritional intervention largely depends on long-term adherence and compliance. However, adherence to dietary interventions in CHD patients is often poor due to barriers such as dialysis time, motivation, and lack of social support [122]. Therefore, CHD patients should be advised on protein options that are easy to prepare, convenient to consume, and have an acceptable taste.

A well-orchestrated lifestyle intervention program combining exercise and nutritional interventions for CHD patients is required to attenuate or even prevent the loss of muscle mass, strength, and functional capacity in this population. For such a multimodal interventional approach to be effective, a (more) personalized supervision of CHD patients provided by a team of healthcare specialists with physical activity and nutritional expertise is required. A close collaboration between nephrologists, physical therapists, and dietitians in both research and clinical care will be essential to improve the health and well-being of the growing number of CHD patients.

## 6. Conclusions

The gradual loss of skeletal muscle mass in CHD patients accelerates after initiation of intermittent HD treatment. Muscle protein breakdown rates in CHD patients are increased, while muscle protein synthesis rates fail to match this increase due to insufficient protein ingestion, amino acid extraction during HD, and the prevalence of anabolic resistance. Protein intake of CHD patients should be increased on dialysis days to compensate for extraction of circulating amino acids during HD and to compensate for the blunted muscle protein synthetic response to feeding in these patients. Implementing structured physical activity in the daily routine of CHD patients represents a feasible strategy to increase the skeletal muscle protein synthetic response to protein ingestion and, as such, to alleviate anabolic resistance. More insight in the impact of protein ingestion and exercise in CHD patients on both dialysis as well as non-dialysis days is required to develop more effective nutritional and exercise intervention programs that can attenuate or even prevent muscle loss in CHD patients.
